# Revalorizing Apple Peel Flour as a Functional Component in Wheat‐Based Cookies: Effects on Nutritional Profile and Quality Characteristics

**DOI:** 10.1002/fsn3.72114

**Published:** 2026-07-18

**Authors:** Isam A. Mohamed Ahmed, Feyza Nur Özsağır, Elfadıl E. Babiker, Mehmet Musa Özcan, Belal M. Mohammed, Nurhan Uslu, Mahmoud Younis, Fahad AlJuhaimi, Kashif Ghafoor, Hesham Al‐Quh

**Affiliations:** ^1^ Department of Food Science & Nutrition, College of Food and Agricultural Sciences King Saud University Riyadh Saudi Arabia; ^2^ Department of Food Engineering, Faculty of Agriculture Selcuk University Konya Turkey; ^3^ Chair of Dates Industry and Technology King Saud University Riyadh Saudi Arabia; ^4^ The School of Agriculture, Food, and Ecosystem Sciences The University of Melbourne Melbourne Victoria Australia

**Keywords:** apple peel flour, bioactive properties, color, flour cookies, polyphenols, sensory evaluation

## Abstract

The physicochemical and bioactive characteristics, fatty acid profile, and sensory attributes (such as odor, taste, and overall acceptability) of cookies produced by mixing 5% and 15% apple peel powder into wheat flour were evaluated. While the L* and b* values of the cookies decreased with cooking, the a* values increased. The L* values of the cookie samples varied between 73.79 (15%) and 85.82 (Control), while the b values were measured between 25.57 (5%) and 28.68 (Control). In addition, the a values of cookies were determined between 0.28 (Control) and 5.21 (15%). While the total phenolic amount of the cookies prepared with the addition of 5% and 15% apple peel powder varied between 18.20 and 46.44 mg/100 g, the total flavonoid amount of the cookies was provided to be between 95.00 mg/100 g and 159.76 mg/100 g. The antioxidant capacities of the cookies varied between 0.15 and 2.35 mmol/kg. The major phenolic compounds in the dried apple peel were identified as catechin, rutin, gallic acid, and hesperidin. The predominant fatty acids of cookies were linolenic, linoleic, oleic, and palmitic acid. Palmitic and oleic acid quantities of cookie oils ranged from 25.07% (5%) and 26.38% (Control) to 4.71% (15%) and 4.89% (5%), respectively. Apple peel incorporation increased the Nutritive Value Index (NVI) of cookie oils from 0.183 (Control) to 0.200 at 5 min, followed by a slight decrease to 0.189 at 15 min. Despite this reduction, NVI values remained above the control, indicating a modest improvement in lipid nutritional quality. The macro element detected in the cookie samples was K in the highest amounts, followed by P, Ca, and Mg in decreasing order. In general, the sensory properties of cookies with 5% apple peel flour concentration had better values than those with 15%.

## Graphical abstract

In this study, the bioactive compounds, phytochemicals, fatty acids, nutrients, and sensory properties of cookies prepared using wheat flour and apple peel powder were investigated.

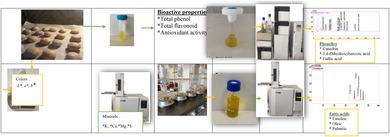

## 1 Introduction

In recent years, the number of studies conducted on the use of foods rich in nutritional and bioactive components in the enrichment of bakery products has increased considerably. In particular, alternative plant sources phytochemicals are gaining increasing importance in this field due to their compatibility with the textural and sensory qualities of bakery products (Föste et al. 2014; Babiker et al. 2020; Özcan 2023). In recent years, due to the growing number of malnourished individuals and the exhaustion of natural resources, the use of large amounts of fruit waste—such as seeds and pulp—to reduce, assess, and improve the sustainability of the food industry has become an increasingly common subject in international scientific research, leading to a rise in related studies. The evaluation of fruit waste is important not only to reduce the waste load in landfills, but also to encourage producers to establish a sustainable economy, as it creates a great economic impact on businesses through handling (Kandemir et al. 2022). Today, bakery goods constitute a fundamental component of human nutrition, and the characteristics of the flours used in their production directly influence the nutritional quality of these products. It has been reported that the nutritional value of bread and similar baked goods made from refined flour is considerably reduced, mainly because the nutrient‐dense germ and fiber‐rich bran fractions of wheat are removed during the milling process (Indrani et al. 2015; Pasqualone et al. 2017). In recent years, alongside rising consumer awareness, demand for healthy and functional foods has also increased. Nevertheless, breads produced from refined flour remain inadequate sources of protein, minerals, essential fatty acids, and dietary fiber. Therefore, research focused on enhancing bakery products with dietary fiber, vitamins, and minerals has gained significant importance. In such enrichment studies, blends derived from protein‐rich foods, particularly plant‐based sources, are commonly utilized (Bastos et al. 2014; Diana et al. 2014). The reason why bakery products are so common is that they are delicious and satisfying, as well as having nutritional values. Bakery products, which are an important part of nutrition, can have different nutritional values depending on their type and preparation method and can support a healthy life when consumed as part of a balanced diet (Anonymous 2020). Apple consumption is highly beneficial for human health because it contains a rich variety of antioxidants, vitamins, and dietary fiber. As a result, regular apple consumption is associated with stronger immune function and improved overall health protection. Overall, apples play an important role in maintaining a balanced and healthy diet (Boyer and Liu 2004; Hyson 2011). The positive health effects associated with apples are largely attributed to their rich composition of phenolic compounds, particularly flavonoids, as well as their significant amounts of vitamin C, antioxidants, and dietary fiber (Boyer and Liu 2004; Tsao et al. 2005). Apple peels are reported to contain 3 to 6 times more flavonoids than apple flesh (Rupasinghe et al. 2010). Apple peel has been reported to have strong antioxidant activity due to its high concentration of phenolic compounds (Drogoudi et al. 2008; Rahman et al. 2010). Antioxidant activity values in the peel are approximately 58% higher than the antioxidant activity found in the flesh (Tsao et al. 2005; Wojdylo et al. 2008; Vieira, Borges, Copetti, Gonzaga, et al. 2009; Vieira, Borges, Copetti, Amboni, et al. 2009). Studies have shown that total phenol and total flavanol contents in various apple peels are higher than in fruit flesh (Vieira et al. 2011). It has been reported that these quantitative fluctuations in apples are mainly due to flavonol glycosides and also to high catechin and chlorogenic acid levels in the peel (Petkovsek et al. 2007; Ravindran et al. 2018). The most abundant phenolic constituents in apple peel and flesh are catechins, procyanidins, phloridzin, phloretin glycosides, caffeic acid and chlorogenic acid (Gornas et al. 2015). It has been reported that the most commonly cultivated apple varieties “Golden Delicious” and “Stark Delicious” have higher levels of phenolic constituents and antioxidant activity (Lacopini et al. 2010). Fruit phenolic composition is influenced by environmental conditions and postharvest factors such as harvesting season, stage of ripeness, and processing methods (McGhie et al. 2005; Lata 2007). Moreover, the cultivar of apple significantly affects its polyphenol profile (McGhie et al. 2005; Scalzo et al. 2005; Khanizadeh et al. 2008). Bakery products, which are consumed globally on a large scale, are often enriched with various edible plant‐derived ingredients. In addition to enhancing their functional properties, such fortification can turn bakery items into functional foods that promote satiety and may contribute positively to human health (Rosell 2016; Yadav et al. 2016; Betoret and Rosell 2019; Aljuhaimi et al. 2024). It has been stated that the use of bioactive compounds or plant edible materials rich in bioactive compounds in the production of bakery products not only increases the nutritional content of bakery products but also their functional properties (Foschia et al. 2013; Fendri et al. 2016; Aljuhaimi et al. 2024). Recently, the use of edible by‐products from the food processing industry in various food products has led to improvements in the nutritional and technological properties of the final products (Bertagnolli et al. 2014; Abreu et al. 2019). The proportion of apple peel relative to the total weight of the fruit is considered to exhibit higher bioactivity than other parts of the apple, particularly because this portion is frequently discarded as waste during apple processing or prior to consumption (Lata and Tomala 2007). In recent years, the decrease in physical activity and changes in eating habits as a result of the changes in people's lifestyles have led to an increase in type 2 diabetes, heart diseases and some chronic diseases. The most important changes in eating habits are the increase in the consumption of “fast food” type rich in rapidly digestible carbohydrates instead of high fiber foods, whole grains, fruits and vegetables. The decrease in dietary fiber amount and the increase in the consumption of foods with a high glycemic index is considered as an important reason for the spread of chronic diseases. Inclusion of high fiber and low glycemic index foods in the diet makes it easier to manage body weight as well as helping glycemic control (Hu et al. 2009). In the production of fruit juice, jam, jelly and marmalade, many parts of the fruit, especially the peel, are disposed of as waste. These wastes can also harm the environment. Since the peels of fruits such as apples, which are directly consumed by cleaning their outer surfaces, are edible, they have recently begun to be used to increase the nutritional properties of various foods, especially bakery products. Therefore, in this study, the peel powder of the Red Delicious apple variety were used in order to enrich the nutritional value and phytochemical content of flour cookies. The objective of this research was to investigate the changes in the physicochemical properties, bioactive components, antioxidant capacity, phenolics, fatty acid composition, lipid indices and sensory properties of wheat flour cookies fortified with apple peel flour at 5% and 15% concentrations.

## 2 Materials and Methods

### 2.1 Materials

**FIGURE 1:**
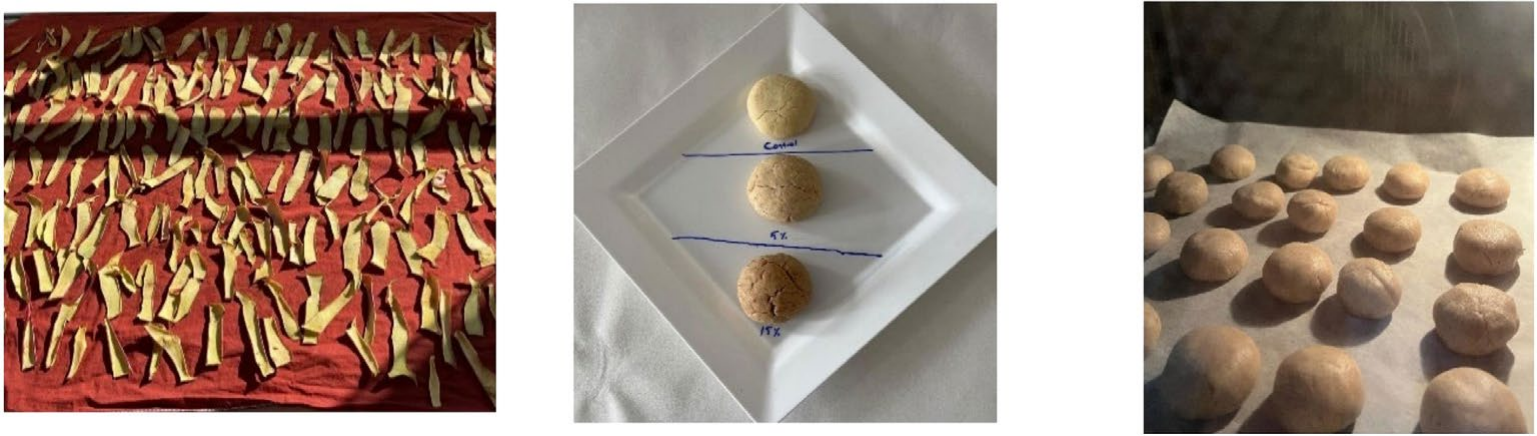
Wheat flour cookies made with apple peel powder.

Five kilograms of “Red Delicious” apple variety was obtained from an apple orchard in Kahramanmaraş province and transferred to the laboratory. After the apples were washed with water, their skins were peeled and dried on trays in the open air. The dried apple peels were powdered to make flour (Figure 1). The cookies were made using wheat flour, butter, and wheat starch. The physicochemical properties of the apple flour sample are presented in Table 1.

**TABLE 1:** Physicochemical and bioactive properties of apple peel used in cookie production.

Physico‐chemical properties		Phenolics (mg/100 g)		Minerals (mg/kg)	
Moisture (%)	7.33 ± 0.43	Gallic acid	24.74 ± 0.43	P	324.53 ± 6.74
Fat (%)	2.10 ± 0.0	Protocatechuic	3.13 ± 0.24	K	7996.17 ± 9.56
L*	67.45 ± 0.46	Chlorogenic acid	7.23 ± 0.07	Ca	677.55 ± 5.78
a*	6.66 ± 0.08	Caffeic acid	2.38 ± 0.14	Mg	475.57 ± 3.18
b*	23.23 ± 0.20	Coumaric acid	0.04 ± 0.01	Fe	24.29 ± 0.90
Total phenolic (mg GAE/100 g)	124.19 ± 0.00	Ferulic acid	3.49 ± 0.73	Zn	7.56 ± 0.013
Total flavonoid (mg/100 g)	800.24 ± 18.09	Cinnamic acid	0.07 ± 0.01	Cu	2.54 ± 0.04
Antioxidant activity (mmol/kg)	7.72 ± 0.00	Catechin	73.51 ± 1.36	Mn	7.63 ± 0.108
		Rutin	26.85 ± 2.38	B	12.66 ± 1.50
		Hesperidin	24.53 ± 2.20		
		Quercetin	3.51 ± 1.08		
		Kaempferol	2.26 ± 0.45		

### 2.2 Methods

#### 2.2.1 Preparation of Cookie Samples

Each dough formula of flour cookies prepared with certain apple peel flour concentrations (5 and 15%) was used: 90 g powdered sugar, 70 mL oil, 125 g butter, and 40 g starch. Apple peel concentration was calculated based on the amount of flour used. The control group was prepared without adding apple peel flour. The cookies were produced in a laboratory setting through hand kneading and shaping. The mixture was kneaded for 10 min and then baked at 170°C for 20 min (Figure 1).

#### 2.2.2 Moisture Contents

The moisture amounts of cookie samples were determined using the KERN & SOHN GmbH infrared moisture analyzer (AOAC 2000).

#### 2.2.3 L*, a*, and b* Values

A Minolta Chroma meter CR 400 (Konica Minolta Inc. Osaka, Japan) was used for analysis of the cookie samples (Pagliarini and Rastelli 1994).

#### 2.2.4 Extraction Procedure

The cookie samples were extracted following the method described by Jakopic et al. (2011). First, 15 mL of methanol was added to 5 g of ground sample, and the mixture was placed in an ultrasonic water bath for 30 min. It was then centrifuged at 6000 rpm for 10 min. After centrifugation, 15 mL of n‐hexane was added, and the mixture was vortexed for 2 min to ensure thorough mixing. The solution was then transferred into a separatory funnel to allow phase separation between methanol and hexane layers. This liquid–liquid extraction step was repeated twice using 10‐mL portions of n‐hexane. Finally, after evaporation, the resulting extract was redissolved in 10 mL of methanol.

#### 2.2.5 Total Phenolic Contents

The total phenolic amount of cookie samples was established using the modified Folin–Ciocalteu assay reported by Yoo et al. (2004). The samples were incubated in the dark at room temperature for 2 h. Then, 5 mL of distilled water was introduced into each sample, and the absorbance was recorded at 725 nm. The total phenolic quantity was established by means of a calibration curve prepared using gallic acid standard solutions of different concentrations.

#### 2.2.6 Total Flavonoid Amounts

The total flavonoid quantity of cookie samples was established using a modified version of the method stated by Dewanto et al. (2002). In this procedure, after preparing the sample, distilled water was added to each test tube to adjust the final volume to 5 mL. The contents of the tubes were then thoroughly mixed to ensure proper homogenization of the reaction mixture. Afterward, the absorbance of the prepared solution was recorded at 510 nm using a spectrophotometer. The total flavonoid amount in the cookie samples was reported as milligrams of catechol equivalents per gram of dry weight (mg CE/g).

#### 2.2.7 Antioxidant Activity Analysis (DPPH)

The antioxidant capacity of the seed extracts was established using the 2,2‐diphenyl‐1‐picrylhydrazyl (DPPH) method according to the procedure reported by Lee et al. (1998). In brief, 0.1 mL of the extract was mixed with 0.9 mL of buffer solution and 2 mL of DPPH solution in test tubes. The mixtures were then vortexed thoroughly to achieve homogeneity. The samples were subsequently kept in the dark for 30 min to enable the reaction to occur. Following incubation, absorbance values were measured at 517 nm Babiker et al. (2020).

#### 2.2.8 Determination of Phenolic Components

A Shimadzu HPLC system fitted with a photodiode array (PDA) detector was employed to detect and measure phenolic compounds in cookie samples. Separation of the compounds was performed using a binary mobile phase made up of 0.05% acetic acid in water (solvent A) and acetonitrile (solvent B). An aliquot of 20 μL of the sample extract was injected and analyzed at a steady flow rate of 1 mL/min, while the column temperature was kept at 30°C. Each analytical run lasted a total of 60 min. Detection of compounds was performed at a wavelength of 280 nm using the PDA detector. The elution was programmed according to a gradient system as follows: from 0 to 0.10 min, 8% B; from 0.10 to 2 min, 10% B; from 2 to 27 min, 30% B; from 27 to 37 min, 56% B; from 37 to 37.10 min, 8% B; and finally from 37.10 to 45 min, the system was maintained at 8% B to re‐equilibrate the column. Each sample required a total runtime of 60 min for complete analysis. The obtained results were expressed as mg/100 g, as reported by Khang et al. (2016).

#### 2.2.9 Determination of Total Fat

The total fat content of cookies was assessed using a Soxhlet extraction method with petroleum ether as the solvent. Prior to extraction, the seeds were milled in a laboratory grinder and passed through a 0.5‐mm mesh sieve. A 10‐g portion of the ground sample was accurately weighed and placed into a Soxhlet extraction thimble. The extraction was performed using petroleum ether at 50°C for a duration of 6 h. Following extraction, the solvent was removed from the obtained miscella using a rotary vacuum evaporator maintained at 50°C. The fat content was then quantified gravimetrically according to the method described by AOАС (2000).

#### 2.2.10 Fatty Acid Composition

Fat esterification was conducted in accordance with the ISO 5509 (ISO 1978) method using n‐hexane and methanolic KOH. Fatty acid methyl esters (FAMEs) were subsequently analyzed using a Shimadzu GC2010 gas chromatograph fitted with a flame ionization detector (FID) and a capillary column.

Device operating conditions:

Detector: 260°C.

Injection block: 260°C.

Carrier gas: Nitrogen.

Total flow rate: 80 mL/min.

Nitrogen flow rate: 1.51 mL/min.

Split ratio: 1/40 mL/min.

Temperature program: hold at 90°C for 7 min, increase by 5°C/min to 240°C, wait at this temperature for 15 min.

#### 2.2.11 Calculation of Lipid Indices of Cookie Fats

The Nutritive Value Index (NVI), Calculated Oxidizability Value (Cox), Oleic Desaturation Ratio (ODR), and Linoleic Desaturation Ratio (LDR) of cookies fortified with apple peels were determined following the methodologies reported by Chen et al. (2016), Fatemi and Hammond (1980), Ulbricht and Southgate (1991), and Pleines and Friedt (1988), respectively.

$$\mathrm{NVI}\;\left(\text{Nutritive Value Index}\right)=\left(C18:0+C18:1\right)/C16:0$$

$$\text{Oxidizability Values}\;\left(\mathrm{Cox}\right):\left[1\;\left(18:1\%\right)+10.3\;\left(18:2\%\right)+21.6\;\left(18:3\%\right)/100\right]$$

ODR=%C18:2+%C18:3/%C18:1+%C18:2+%C18:3

LDR=%C18:3/%C18:2+%C18:3

#### 2.2.12 Mineral Analysis of Cookie Samples

After 5 mL 65% HNO_3_ and 2 mL 30% H_2_O_2_ were added into each powdered cookie sample, the solution was incinerated at 210°C and 200 PSI in a closed microwave system. After pretreatments, the quantitative values of the elements were measured with ICP‐OES equipment (Tošic et al. 2015).

#### 2.2.13 Sensory Properties

The score test was selected to evaluate the sensory characteristics of the cookie samples. In this context, 18 semi‐trained panelists were instructed to assign numerical ratings ranging from 1 to 5. All sample groups were simultaneously served to the panelists in identical quantities and with a uniform presentation. The sensory evaluation scale was defined as follows: 1 indicated “very bad,” 2 “bad,” 3 “medium,” 4 “good,” and 5 “very good.”

### 2.3 Statistical Analysis

Analysis of variance (ANOVA) was performed using JMP software version 9.0. The results are presented as mean ± standard deviation (calculated with MSTAT C) for different independent roasting temperatures. Differences among the cookie samples were evaluated using Duncan's Multiple Range Test at a significance level of *p* < 0.05.

## 3 Results

### 3.1 The Physicochemical and Bioactive Properties of Wheat Flour Cookies Made by Adding Apple Peel Powder

The physicochemical and bioactive properties of flour‐based cookies enriched with varying levels of apple peel powder are shown in Table 2. The amount of apple peel powder incorporated into the cookie dough significantly influenced both the color attributes and the bioactive profile of the final product. These variations can likely be attributed to the inherent composition of apple peel, as well as enzymatic and non‐enzymatic reactions occurring during baking due to heat exposure. Moisture quantity of the cookies ranged from 4.88% in the control sample to 6.45% in the 15% apple peel formulation. Oil content was found to vary between 27.30% (control) and 29.70% (15%). The addition of apple peel appeared to enhance moisture retention while also contributing to a slight increase in fat content, possibly due to the lipid components present in the peel. Significant changes in color parameters were observed during baking (*p* < 0.05). The L* (lightness) and b* (yellowness) values decreased with increasing apple peel concentration, whereas the a* (redness) values increased. Specifically, L* values ranged from 85.82 (control) to 73.79 (15%), while b* values varied between 28.68 (control) and 25.57 (5%). The a* values increased from 0.28 (control) to 5.21 (15%). Total phenolic content and flavonoid amount of the cookies were reported to range from 18.20 (5%) to 46.44 mg GAE/100 g (15%) and from 95.00 (control) to 159.76 mg/100 g (15%), respectively (Table 2). Furthermore, antioxidant activity values were found to range between 0.15 (control) and 2.35 mmol/kg (15%). Overall, increasing the concentration of apple peel powder enhanced the bioactive properties of the cookies.

**TABLE 2:** Physicochemical and bioactive properties of flour cookies enriched with apple peel powder.

Apple peel powder (%)	Moisture (%)	L*	a*	b*
Control	4.88 ± 0.21c*	85.82 ± 0.50a	0.28 ± 0.38c	28.68 ± 0.11a
5%	5.80 ± 0.04b	78.66 ± 0.28b	1.78 ± 0.48b	25.57 ± 0.47b
15%	6.45 ± 0.07a	73.79 ± 0.51b	5.21 ± 0.33a	25.78 ± 1.02b

Apple peel powder (%)	Oil (%)	Total phenol (mgGAE/100 g)	Total flavonoid (mg/100 g)	Antioxidant activity (mmol/kg)
Control	27.30 ± 0.28a	18.20 ± 1.59a	95.00 ± 20.75a	0.15 ± 0.00a
5%	27.60 ± 0.57a	23.78 ± 0.31b	101.67 ± 2.97b	0.74 ± 0.01b
15%	29.70 ± 0.28b	46.44 ± 1.64c	159.76 ± 7.87c	2.35 ± 0.01c

* Standard deviation, and values followed by different letters in each column are significantly different at *p* < 0.05.

### 3.2 Phenolic Compounds of Wheat Flour Cookies Made by Adding Apple Peel Powder

The amounts of phenolic compounds in flour cookies prepared by adding apple peel powder at different concentrations are shown in Table 3. The dominant phenolic compounds of the cookies were gallic acid and kaempferol. The hesperidin value of the cookies increased gradually with the increase in apple peel flour addition. While protocatechuic acid was detected in apple peel, it was not detected in the cookie samples. In addition, chlorogenic acid, coumaric acid, and ferulic acid were detected only in cookies with apple flour added. In addition, caffeic acid was determined in the control sample, but it was not detected in apple peel cookies. Gallic acid and kaempferol contents of cookies were determined as 2.92 (15%) and 7.04 mg/100 g (Control) to 0.15 (15%) and 0.16 (control and 5%), respectively. In general, the phenolic constituents of cookies enriched with apple peel decreased when compared to the phenolic compounds of control and apple peel. The highest amounts of phenolic compounds were found to be almost close to each other in cookies produced at both concentrations. Caffeic acid, coumaric acid, ferulic acid, catechin, and rutin were high in 15% concentration cookies, while other phenolic compounds were high in 5% apple peel fortified cookies. The rutin and quercetin contents of cookies were assayed to be between 0.28 (5%) and 1.70 (control) to 0.74 (15%) and 1.58 mg/100 g (control), respectively.

**TABLE 3:** Phenolic compound content of flour cookies enriched with apple peel powder.

Phenolic compounds (mg/100 g)	Control	5%	15%
Gallic acid	7.04 ± 0.71c*	4.31 ± 0.72b**	2.92 ± 0.23a
Protocatechuic acid	—**	—	—
Chlorogenic acid	—	0.11 ± 0.01a	0.29 ± 0.02b
Caffeic acid	0.03 ± 0.00	—	—
Coumaric acid	—	0.03 ± 0.00a	0.09 ± 0.03b
Ferulic acid	—	0.05 ± 0.01a	0.29 ± 0.03b
Cinnamic acid	0.08 ± 0.02b	0.08 ± 0.03b	0.06 ± 0.02a
Catechin	0.71 ± 0.19b	0.39 ± 0.16a	2.51 ± 0.89c
Rutin	1.70 ± 0.55c	0.28 ± 0.05a	0.81 ± 0.37b
Hesperidin	0.27 ± 0.10a	1.03 ± 0.18c	0.54 ± 0.04b
Quercetin	1.58 ± 0.08c	0.97 ± 0.12b	0.74 ± 0.19a
Kaempferol	4.76 ± 1.01c	2.17 ± 0.26b	0.75 ± 0.18a

*Note:* —, not determined.

* Standard deviation, and values followed by different letters in each row are significantly different at *p* < 0.05.

** Not determined.

### 3.3 The Fatty Acid Profile of Fats Extracted From Cookies Enriched With Apple Peel Flour

The fatty acid profiles of the fats obtained from cookies fortified with apple peel flour at two different concentrations are presented in Table 4. The highest abundant fatty acid in apple peel oil was behenic acid (75.30%), followed by linolenic (8.65%), linoleic (6.81%), and palmitic acid (5.69%) in decreasing order. The predominant fatty acids of cookie fats were linolenic, linoleic, oleic, and palmitic acid. Palmitic and oleic acid amounts of cookie fats ranged from 25.07% (5%) and 26.38% (control) to 4.71% (15%) and 4.89% (5%), respectively. Besides, the amounts of linoleic and linolenic fatty acids in cookie fats were characterized to be between 32.63% (15%) and 34.17% (5%) to 35.41% (5%) and 36.56% (15%), respectively.

**TABLE 4:** Fatty acid composition of the oils extracted from flour cookies enriched with apple peel powder.

Fatty acids (%)	Control	5%	15%
Palmitic	26.38 ± 1.45a*	25.07 ± 3.01bc	25.66 ± 0.31b
Stearic	0.11 ± 0.01a	0.12 ± 0.03b	0.13 ± 0.00c
Oleic	4.72 ± 0.12a	4.89 ± 0.30b	4.71 ± 0.01a
Linoleic	32.76 ± 0.65b	34.17 ± 1.47a	32.63 ± 0.04b
Arachidic	0.16 ± 0.01a	0.16 ± 0.01a	0.17 ± 0.00b
Linolenic	35.71 ± 0.68a	35.41 ± 1.23ab	36.56 ± 0.28c
Behenic	0.16 ± 0.00a	0.16 ± 0.02a	0.15 ± 0.00b

* Standard deviation, and values followed by different letters in each row are significantly different at *p* < 0.05.

### 3.4 Lipid Index Values of the Fats Extracted From Cookie Enriched by the Apple Peels

Table 5 presents the lipid index values of fats obtained from cookies enriched with apple peel. The Nutritive Value Index (NVI) of the extracted fats showed an increase from 0.183 in the control sample to 0.200 after 5 min of apple peel incorporation, indicating an improvement in the nutritional quality of the lipid fraction. Although a slight reduction was observed at 15 min (0.189), the value still remained above that of the control group, suggesting that the enhancement effect was largely maintained. Similarly, the Oxidizability Value (Cox) exhibited a gradual upward trend, rising from 111.34 in the control to 112.17 at 5 min and further to 113.05 at 15 min. This increase reflects a higher degree of unsaturation in the lipid profile, which is typically associated with greater theoretical susceptibility to oxidative degradation. In contrast, the Oleic Desaturation Ratio (ODR) remained almost constant across all samples, ranging narrowly between 0.934 and 0.936. This stability indicates that the oleic acid fraction was not significantly affected by the addition of apple peel or processing time. On the other hand, the Linoleic Desaturation Ratio (LDR) showed minor fluctuations. It decreased slightly at 5 min (0.509) compared to the control (0.513), but increased to 0.528 at 15 min. This pattern suggests that longer incorporation times may contribute to better retention or availability of linoleic acid within the lipid fraction.

**TABLE 5:** Lipid index values of the fats extracted from flour cookies enriched with apple peel.

Lipid index	Control	5 min	15 min
Nutritive Value Index (NVI)	0.183	0.200	0.189
Oxidizability Value (Cox)	111.34	112.17	113.05
Oleic Desaturation Ratio (ODR)	0.936	0.934	0.936
Linoleic Desaturation Ratio (LDR)	0.513	0.509	0.528

### 3.5 Mineral Contents of Wheat Flour Cookies Enriched With Apple Peel Powder

The mineral contents of cookie samples enriched with apple peel flour are given in Table 6. The macroelements detected in the cookie samples were predominantly K, followed by P, Ca, and Mg in descending order. Potassium (K) and phosphorus (P) contents ranged from 887.75 mg/kg (15%) to 2352.21 mg/kg (5%), and from 193.41 mg/kg (15%) to 459.19 mg/kg (5%), respectively. Calcium (Ca) levels varied between 100.95 mg/kg (15%) and 266.05 mg/kg (control), while magnesium (Mg) ranged from 55.32 mg/kg (15%) to 168.73 mg/kg (5%). Among the microelements, boron (B) was present in the highest concentration, followed by Fe, Zn, Cu, and Mn in decreasing order. Boron content ranged from 26.86 mg/kg (control) to 29.32 mg/kg (5%). Iron (Fe) and zinc (Zn) levels ranged from 9.25 mg/kg (control) to 13.47 mg/kg (15%), and from 5.12 mg/kg (control) to 7.53 mg/kg (15%), respectively.

**TABLE 6:** Mineral contents of wheat flour cookies enriched with apple peel powder (mg/kg).

Samples	P	K	Ca	Mg
Control	443.42 ± 3.66 B*	1394.66 ± 61.06 B	266.05 ± 2.28 A	111.76 ± 2.24 B
5%	459.19 ± 6.01 A	2352.21 ± 4.19 A	219.21 ± 1.66 B	168.73 ± 20.85 A
15%	193.41 ± 5.48 C	887.75 ± 6.45 C	100.95 ± 2.30 C	55.32 ± 2.86 C

Samples	Fe	Zn	Cu	Mn	B
Control	9.25 ± 0.87 A	5.12 ± 0.063 BC	5.35 ± 0.17 A	2.55 ± 0.047 B	26.86 ± 1.68 AB
5%	13.47 ± 1.08 B	5.69 ± 0.143 B	5.06 ± 0.02 B	2.45 ± 0.021 BC	29.32 ± 2.19 A
15%	5.23 ± 0.05 C	7.53 ± 0.013 A	2.68 ± 0.11 C	5.04 ± 0.012 A	29.23 ± 1.71 A

* Standard deviation, and values followed by different letters in each row are significantly different at *p* < 0.01.

### 3.6 Sensory Properties of Cookies Prepared With Apple Peel Flour

Sensory properties of cookies prepared with apple peel flour at different concentrations are depicted in Table 7. Taste and odor scores of cookies were evaluated between 4.33 (15%) and 4.83 (control) to 4.67 (5% and 15%) and 4.83 (control), respectively. Color scores of cookies varied between 4.17 (15%) and 5.00 (control), while texture values of cookies were evaluated between 6.67 (15%) and 4.83 (control and 5%).

**TABLE 7:** Sensory properties content of flour cookies enriched with apple peel powder.

Apple peel powder	Flavor	Odor	Color	Texture	General appreciation
Control	4.83 ± 0.41a*	4.83 ± 0.41a	5.00 ± 0.00a	4.33 ± 0.82c	4.83 ± 0.41a
5%	4.67 ± 0.52b	4.67 ± 0.52b	4.83 ± 0.41b	4.67 ± 0.52a	4.83 ± 0.41a
15%	4.33 ± 0.52c	4.67 ± 0.82b	4.17 ± 0.75bc	4.50 ± 0.84b	4.67 ± 0.52b

* Standard deviation, and values followed by different letters in each column are significantly different at *p* < 0.05.

## 4 Discussion

Although the incorporation of apple peel did not lead to a statistically significant change in the b* color values of the cookies, a slight reduction was still observed when compared with the control sample. This reduction may be attributed to the formation of both enzymatic and non‐enzymatic reaction products during baking, including processes such as caramelization and the Maillard reaction, which can influence color development. In related literature, Güzel and Akpınar (2019) reported that the fat content of various fruit and vegetable peels ranged between 1.62% and 3.61%, indicating considerable variability depending on the source material. Similarly, Karabudak et al. (2013), in their investigation of the nutritional composition of different cookie types, identified a wide range of fat contents, with the lowest value (22.7%) observed in grape cookies and the highest value (38.3%) in German cookies. Furthermore, in a study examining cookies enriched with varying levels of lemon peel powder (2%, 4%, 6%, 8%, and 10%), it was found that fat content differed according to the substitution level. The highest fat content (18.03%) was recorded in the sample containing 10% lemon peel powder, whereas the lowest value (17.24%) was observed in the sample with 2% lemon peel powder addition (Güneruz 2022). Significant changes were observed in the color parameters of the cookies during baking (*p* < 0.05). The L* and b* values decreased with baking, whereas the a* values showed an increase. The L* values ranged from 73.79 (15%) to 85.82 (Control), while the b* values varied between 25.57 (5%) and 28.68 (Control). Moreover, the a* values were found to be between 0.28 (Control) and 5.21 (15%). Dirim et al. (2023) reported that the maximum L* value, indicating brightness, of apple peel powder reached 70.70 when the samples were dried in a convection oven at 120°C. In the same study, the highest a* value, representing the red‐green color axis, was found to be 9.68 when microwave drying was applied at 540 W. Moreover, the greatest b* value, reflecting the yellow‐blue color dimension, was recorded as 36.90 under convection oven drying at 120°C. Overall, the findings indicated that drying methods such as oven drying, freeze drying, and drum drying generally led to reductions in L* (lightness) and b* (yellowness–blueness) values compared to the control samples. In contrast, the a* (redness–greenness) values showed an increasing trend after the drying processes, suggesting a shift toward more reddish tones in the dried apple peel powders (Henríquez et al. 2013).

In a previous investigation, the highest level of total phenolic compounds was reported in cookie formulations containing 6% lemon peel powder, reaching 346.69 mg/100 g, whereas the lowest value was observed in the control cookies without any addition (261.78 mg/100 g) (Güneruz 2022). These findings are consistent with the general trend reported in the literature that fruit peels tend to be richer in phenolic compounds than their corresponding edible flesh. For instance, Drogoudi et al. (2008) demonstrated that apple peel contains a higher concentration of total phenolics compared to peeled apple tissue. Similarly, San and Mazı (2023) reported that dried lemon peel exhibits substantial phenolic content, ranging from 554 to 1731 mg GAE/100 g (dry weight basis), highlighting its strong bioactive potential. In agreement with this, Glibowski and Popiolek‐Kalisz (2023) showed that apple peel possesses greater antioxidant activity than apple flesh, further emphasizing the functional superiority of peel fractions. Vieira et al. (2011) also evaluated different apple varieties and found that phenolic content (mg GAE/100 g fresh weight) varied significantly depending on cultivar and tissue type. In fruit flesh, values ranged from 128.3 mg/100 g (Golden Delicious) to 212.0 mg/100 g (Epagri‐F5P283), while in the peel, much higher levels were recorded, ranging from 304.6 mg/100 g (Golden Delicious) to 712.6 mg/100 g (Catarina). In addition, total phenolic and flavonoid contents in apple peels were reported to vary widely across cultivars, with values spanning from 304.66 mg CAE/100 g (Golden Delicious) up to 712.65 mg CAE/100 g (Catarina), while the lowest flavonoid content was observed in Epagri‐SJ11 at 32.42 mg CAE/100 g (Vieira et al. 2011). Supporting these findings, Chinnici et al. (2004) reported that antioxidant activity measured by the DPPH assay in apple peel was approximately 2.5 times higher than in apple flesh. Another study further confirmed that the peel fraction of apples exhibits considerably higher antioxidant capacity than the pulp. Among different apple cultivars, Starkrimson was identified as having the highest antioxidant activity and phenolic concentration in the peel (35.7 and 19.9 mg/g, respectively), followed by Jonagored, Mutsu, Fyriki, and Fuji varieties. It has been reported that among different apple varieties, the peels of Golden Delicious and Granny Smith exhibit the lowest levels of antioxidant capacity (Karaman 2008). In another investigation, Deng et al. (2012) evaluated the antioxidant potential of red and green apple peels using the FRAP assay, reporting values of 10.46 and 11.33 μmol Fe(II)/g, respectively, while the TEAC method yielded corresponding values of 14.89 and 15.49 μmol Trolox equivalents (TE)/g. Similarly, Vieira, Borges, Copetti, Amboni, et al. (2009) characterized the total antioxidant activity of peels from three apple cultivars and found it ranged between 2062.86 and 2099.13 μmol Trolox equivalents per 100 g. In a later study, Vieira et al. (2011) further demonstrated that antioxidant activity in apple peel samples varied considerably among cultivars, with values spanning from 1004.0 μmol TEAC/100 g in Golden Delicious to 3877.73 μmol TEAC/100 g in the Catarina variety. In addition to apple‐based studies, Aljuhaimi et al. (2024) investigated the effect of incorporating chufa flour into cookies and observed that antioxidant activity increased with higher substitution levels, reaching a maximum value of 0.74 mmol/kg at 100% chufa flour addition, whereas the lowest value (0.57 mmol/kg) was recorded in the control samples without chufa flour. Furthermore, Özcan et al. (2023) reported that coriander‐enriched bread samples exhibited notable increases in both total phenolic and flavonoid contents. Total phenolic values ranged from 43.13 mg GAE/100 g in the control sample to 64.01 mg GAE/100 g in bread containing 30% coriander, while flavonoid content increased from 72.62 mg/100 g in the control to 130.71 mg/100 g at the same enrichment level. Overall, it can be observed that the physicochemical and bioactive properties of cookies enriched with apple peel flour differ from previously reported literature values; however, these findings are consistent with the general trend that the incorporation of fruit and plant‐based by‐products enhances the nutritional and functional properties of bakery products.

During baking, the levels of rutin and quercetin in cookies were found to decline as the proportion of apple peel increased. This reduction can be attributed to the thermal degradation of phenolic compounds, which are sensitive to heat processing conditions. Previous research supports the strong phenolic profile of apple peel compared with other parts of the fruit. For instance, Demirci and Erdoğan (2014) reported that apple peels contain approximately five times more polyphenols than the flesh, accounting for nearly 46% of the fruit's total polyphenolic content. They also emphasized that quercetin is predominantly located in the peel, while flavonols are essentially absent in peeled apple tissue. In addition, rutin (identified as quercetin rutinoside) has been detected in apple peels according to several studies (Demirci and Erdoğan 2014). Similarly, Karaman (2008) demonstrated that while apple flesh contains compounds such as procyanidins, catechin, chlorogenic acid, caffeic acid, and phloretin derivatives, the peel additionally contains rutin and various flavonol glycosides, highlighting its richer phenolic diversity. Supporting this, Volz and McGhie (2011) found that concentrations of key phenolics in the peel—such as phloridzin, chlorogenic acid, and quercetin—can be 100 to 250 times higher than those in the inner parts of the apple. Massias et al. (2015) also confirmed that apple peel contains significantly higher total polyphenol levels, particularly quercetin, chlorogenic acid, and phloridzin, compared to both the core and pulp. In related food matrix studies, Aljuhaimi et al. (2024) identified kaempferol, gallic acid, cinnamic acid, and 3,4‐dihydroxybenzoic acid as major phenolic constituents in cookie formulations, with the highest recorded concentrations being 33.88, 5.02, 2.00, and 1.79 mg/100 g, respectively. Likewise, Kandil (2024) reported comparable phenolic profiles in cookie samples, although some variation in compound concentrations was observed. Furthermore, Özcan et al. (2023) showed that bread samples were particularly rich in gallic acid, 3,4‐dihydroxybenzoic acid, catechin, and rutin, followed by caffeic acid, syringic acid, and p‐coumaric acid in decreasing order of abundance. These observed differences can likely be attributed to variations in baking conditions as well as the phenolic compound profile of the plant‐based materials incorporated into the bakery formulations. In addition, the differences between the fatty acid composition of the cookie samples and that of apple peel oil may stem from the type of oil and other ingredients used during cookie production, which can significantly alter lipid profiles. It was also noted that behenic acid was not detected as being transferred from the apple peel into the cookie samples. This absence may be explained by the possible thermal degradation or structural breakdown of behenic acid during the baking process, which could prevent its retention or transformation within the final product matrix. Among the fatty acids identified, linoleic acid was found in the highest proportion in the oil extracted from cookies containing 5% apple peel, while linolenic acid was predominant in those containing 15% apple peel. Furthermore, the relatively high levels of palmitic acid observed in the cookie samples are likely associated with the use of hydrogenated oils in the formulation, which typically contribute higher saturated fatty acid content. From a nutritional perspective, the presence of substantial amounts of linoleic and linolenic acids is particularly important, as these are essential fatty acids that provide recognized health benefits. Supporting these findings, Aljuhaimi et al. (2024) reported that cookie oils produced by substituting wheat flour with different levels of chufa (0%, 25%, 50%, 75%, and 100%) showed dominant fatty acids such as oleic acid (42.33% at 100% substitution), linoleic acid (32.94% at 0%), and palmitic acid (26.16% at 0%). The discrepancies between their results and the present findings may be explained by differences in the fatty acid composition of ingredients such as butter and apple peel oil used in cookie preparation. Overall, the incorporation of apple peel appears to enhance the nutritional profile of the extracted oil by increasing the degree of unsaturation. Moreover, the natural antioxidant compounds present in apple peel may also help reduce oxidative instability, even in cases where higher Cox values are observed. Oleic acid represents the major fatty acid present in olives and demonstrates relatively high stability when subjected to moderate processing conditions (Kiritsakis and Markakis 1987).

Consistent results have also been observed in research investigating the impact of technological treatments on olive oil lipid fractions, where the LDR (linoleic desaturation ratio) was found to remain largely unchanged (Baccouri 2008). In the case of sesame oil, the average ODR (oleic desaturation ratio) has been reported as 0.5, while the average LDR is approximately 0.01 (Mondal et al. 2010). Furthermore, studies on sesame oil extracted from seeds roasted under various environmental conditions have shown calculated oxidizability values ranging between 4.97 and 5.06, with corresponding ODR values varying from 0.5143 to 0.5172 and LDR values from 0.0115 to 0.0136 (El‐Beltagi et al. 2022). Overall, COX (calculated oxidizability) scores across different vegetable oils tend to show only slight variation, suggesting that these oils generally possess a considerable resistance to oxidative degradation (Biglar et al. 2012). Olive oil, in particular, is characterized by a high level of unsaturation, which is reflected in its ODR and LDR indices and indicates a notable presence of unsaturated fatty acids, especially linolenic acid. Since lipid oxidation and fatty acid saturation levels are commonly assessed using COX values or the saturated‐to‐unsaturated (S/P) ratio, it is generally observed that oils with a higher S/P ratio exhibit greater oxidative and fatty acid stability compared to those with lower ratios (Sinanoglou et al. 2013).

Cookies enriched with 5% apple peel showed higher Fe and B contents compared to both the control samples and cookies fortified with 15% apple peel flour. This finding suggests that apple peel is not particularly rich in macro elements, and therefore, incorporating it in high proportions into cookie formulations may not be suitable. In contrast, Zn levels in cookies containing only apple peel increased progressively with rising enrichment levels, which aligns with the relatively high Zn concentration naturally present in apple peel. Regarding other mineral components, P content in the cookie samples ranged from 1630.45 mg/kg (1.5% formulation) to 1859.20 mg/kg (control), while K content varied between 901.22 mg/kg (3.0%) and 1023.61 mg/kg (1.5%) (Özcan et al. 2023). Similarly, Ca and Mg levels in cookies enriched with mahaleb seed flour were reported to range from 1024.45 mg/kg (control) to 1215.88 mg/kg (4.5%) for calcium, and from 251.36 mg/kg (3.0%) to 339.32 mg/kg (4.5%) for magnesium, respectively (Özcan et al. 2023). In addition, Fe concentrations in cookies fortified with mahaleb seed flour varied between 5.37 mg/kg (control) and 6.74 mg/kg (1.5%), while Mn levels were observed to range from 4.58 mg/kg (1.5%) to 5.24 mg/kg (4.5%). Zn content in the same samples changed from 6.26 mg/kg in the control group to 8.28 mg/kg at 1.5% fortification (Özcan et al. 2023). When comparing the mineral composition of apple peel–enriched cookies with values reported in the literature, some variations were observed. These differences are likely attributable to the composition of the wheat flour used, as well as the added bread crust. Overall, these mineral‐rich cookies may help contribute to the dietary mineral intake of consumers.

With the increase in apple flour concentration, a decrease was observed in taste, odor, and color values of cookies, while an increase was observed in texture values. In the study conducted by Nakov et al. (2020), the addition of apple peel powder to wheat flour cookies did not change the physical properties of the products and improved the sensory quality of the products. The addition of 24% apple peel powder gave the best results in terms of appearance, internal structure, texture, and (together with cookies with 16% and 32% apple peel powder) taste. In general, the addition of 24% apple peel powder gave the best cookies with an average score of 4.4 out of 5 (Nakov et al. 2020). According to taste scores, the sample with 4% lemon peel powder added received the highest score (5.49), while the sample with 10% lemon peel powder added received the lowest score (4.99) (Güneruz 2022). In general, sensory properties of cookies with 5% apple peel flour concentration had better values than those with 15%. This situation kept the general appreciation of cookies with 5% concentration high. Although the sensory properties of the cookie with 5% concentration are close to the control, it may be preferred because it increases the phytochemical components of the cookie.

## 5 Conclusion

When the results of the study are examined, it is revealed that dried apple peel powder is a valuable waste that can be used in food enrichment and that by using apple peel powder in addition to flour in the cookie formulation, cookies can be improved with components that are beneficial to health, along with an increase in phenolic substances and antioxidant capacities. No significant changes were monitored in the fatty acid composition of cookies with the addition of apple peel. It is thought that the possible differences in the fatty acid composition of cookie oils are due to the butter used. According to the sensory analysis results, it was observed that with the increase in apple peel powder in cookies, the texture score of cookies increased; taste, color and general appearance scores decreased. With the use of apple peel in food enrichment in the food industry, both the damage to the environment caused by waste can be reduced by recycling apple peels and apple peels can be added to the economy.

## Author Contributions

**Isam A. Mohamed Ahmed:** conceptualization, writing – review and editing. **Hesham Al‐Quh:** validation, data curation, writing – review and editing. **Mahmoud Younis:** software, validation, writing – review and editing. **Fahad AlJuhaimi:** validation, methodology, data curation, software, visualization, writing – review and editing. **Feyza Nur Özsağır:** methodology, formal analysis, writing – review and editing. **Belal M. Mohammed:** data curation, writing – review and editing. **Elfadıl E. Babiker:** data curation, writing – review and editing. **Kashif Ghafoor:** methodology, data curation, writing – review and editing. **Nurhan Uslu:** formal analysis. **Mehmet Musa Özcan:** investigation, formal analysis, methodology, writing – original draft, writing – review and editing.

## Funding

This study was funded (ORF‐2026‐1074) by King Saud University, Riyadh, Saudi Arabia.

## Disclosure

All authors have read and approved the final version of the manuscript. Mehmet Musa Özcan had full access to all of the data in this study and takes complete responsibility for the integrity of the data and the accuracy of the data analysis. We confirm that none of the cited references have been retracted.

## Ethics Statement

This study on cookie with apple peel was conducted exclusively using plant materials and did not involve any human participants, human data, animals, or animal‐derived experimental procedures. Therefore, ethical approval from a human or animal ethics committee was not required for this research. All experimental procedures were carried out in accordance with relevant institutional and scientific guidelines for plant‐based studies.

## Consent

The article was prepared with the consent of each author, and author contributions were made. The purpose and nature of the study were explained in writing to all authors, and I had the opportunity to ask questions about the study.

## Conflicts of Interest

The authors declare no conflicts of interest.

## Data Availability

The data that support the findings of this study are available from the corresponding author upon reasonable request.
